# Chagas Disease, an Endemic Disease in the United States 

**DOI:** 10.3201/eid3109.241700

**Published:** 2025-09

**Authors:** Norman L. Beatty, Gabriel L. Hamer, Bernardo Moreno-Peniche, Bonny Mayes, Sarah A. Hamer

**Affiliations:** University of Florida College of Medicine, Gainesville, Florida, USA (N.L. Beatty); Emerging Pathogens Institute, University of Florida, Gainesville (N.L. Beatty); Texas A&M University, College of Agriculture and Nature Resources, College Station, Texas, USA (G.L. Hamer); University of California, Berkeley, California, USA (B. Moreno-Peniche); Texas Department of State Health Services, Austin, Texas, USA (B. Mayes); Texas A&M University College of Veterinary Medicine and Biomedical Sciences, College Station (S.A. Hamer)

**Keywords:** Chagas disease, *Trypanosoma cruzi*, parasites, vector-borne infections, kissing bug, triatomine, endemicity, autochthonous, United States

## Abstract

Chagas disease, caused by *Trypanosoma cruzi* parasites, is considered endemic to 21 countries in the Americas, excluding the United States. However, increasing evidence of *T. cruzi* parasites in the United States in triatomine insects, domestic animals, wildlife, and humans challenges that nonendemic label. Several triatomine species are common in the southern United States, where they transmit *T. cruzi* and invade human dwellings. Wildlife, captive animals, and companion animals, especially dogs, are commonly infected with *T. cruzi* parasites in this region and serve as reservoirs. Autochthonous human cases have been reported in 8 states, most notably in Texas. Labeling the United States as non–Chagas disease–endemic perpetuates low awareness and underreporting. Classification of Chagas disease as endemic, in particular as hypoendemic, to the United States could improve surveillance, research, and public health responses. Acknowledging the endemicity of Chagas disease in the United States is crucial for achieving global health goals.

Chagas disease, or American trypanosomiasis, is caused by the parasitic protozoan *Trypanosoma cruzi*, which is transmitted through congenital, oral, and vectorborne routes; vectorborne infections result from contact with the feces of infected triatomine insects (kissing bugs). The World Health Organization (WHO) and Pan American Health Organization highlight 21 countries in the Americas to which Chagas disease is endemic (https://www.who.int/publications/i/item/9789240010352; https://www.paho.org/en/topics/chagas-disease), excluding the United States. As a result, the United States is often labeled as nonendemic, and this designation permeates the scientific literature (*1*,*2*), the Centers for Disease Control and Prevention (CDC) website (https://www.cdc.gov/chagas/index.html), the media, pest professional websites, and the general community of researchers and physicians (*3*,*4*). In this article, we review a body of evidence establishing the robust presence of *T. cruzi* parasites in the United States, not only among insect vectors, wildlife, and domestic animals but also among humans without travel histories who are assumed to be locally infected. Through revisiting definitions of endemicity, we conclude that sufficient evidence exists to support the inclusion of the United States as an endemic country for Chagas disease.

## Robust Sylvatic Cycles of *T. cruzi* Parasites in the United States

**Figure 1:**
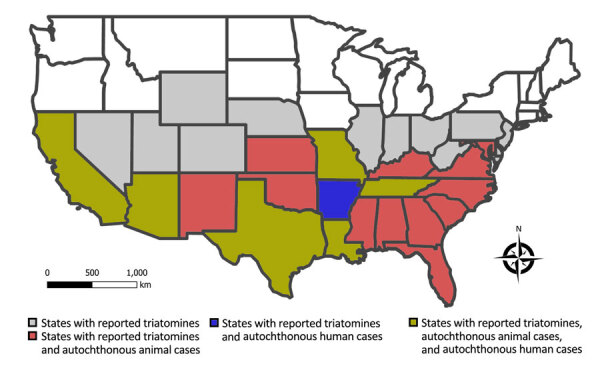
US states with reported wild, domestic, or captive animals exposed to *Trypanosoma cruzi* locally; states with reported autochthonous human Chagas disease; and all states with reported triatomines in assessment of Chagas disease as endemic to the United States.

In the United States, triatomines are commonly known as kissing bugs. The blood-sucking insects occur naturally in the southern half of the country and have been identified in 32 states (https://www.cdc.gov/chagas/index.html) (*5*) (Figure 1). Although available data are inadequate to prove that triatomines are increasing in geographic distribution or abundance, largely owing to a lack of standardized surveillance over time, triatomines are increasingly recognized because of frequent encounters with humans in the domestic and peridomestic habitat and increased research attention (*6*). Invasion into homes, human bites, subsequent allergic reactions or exposure to *T. cruzi* parasites, and increasing frequency of canine diagnoses have led to growing public awareness (*7*–*10*). Of all 11 triatomine species found in the United States, 9 have been found to be naturally infected with *T. cruzi* (*9*,*11*). Of those, 4 species (*Triatoma sanguisuga*, *T. gerstaeckeri*, *T. protracta*, and *T. rubida*) are commonly found in human dwellings, raising concern for increased opportunity for vectorborne transmission to humans (*7*,*8*,*12*,*13*). Although triatomine colonization (defined as the presence of flightless immature nymphal stages in the domicile) occurs in the United States (*12*,*14*), metrics of colonization are lower than those observed in Chagas disease–endemic communities in rural Latin America. Numerous investigations of triatomines in the United States have revealed they harbor *T. cruzi* parasites; infection prevalence ranges from 30% to >50% (*15*,*16*). *Triatoma sanguisuga* and *T. protracta* kissing bugs have the largest overall distribution within the United States, but the *T. gerstaeckeri* kissing bug appears to be the most common species in domestic settings in Texas and is likely responsible for transmission resulting in locally acquired *T. cruzi* infection in dogs and humans (*9*).

*T. cruzi* infections among sylvatic and peridomestic mammalian reservoirs have been documented in >17 states in the southern United States (Figure 1) (Appendix) and include species such as woodrat (*Neotoma* spp.), Virginia opossum (*Didelphis virginiana),* raccoon (*Procyon lotor),* nine-banded armadillo (*Dasypus novemcinctus*), striped skunk (*Mephitis mephitis*), and coyote (*Canis latrans*) (*6*). Infection prevalence among some wild mammal populations can be as high as >50%, and parasitemias are considered high enough to infect triatomines, thus these mammals function as reservoir hosts (*6*,*17*). *T. cruzi* discrete typing units I and IV have been consistently identified in wild and domestic reservoir species (*6*,*9*,*18*); additional discrete typing units have been detected using deep-sequencing methods (*19*). Among wildlife reservoirs in the United States, Virginia opossums can possess the unique feature of harboring *T. cruzi* parasites within the anal gland and anal gland secretions, and vertical transmission from infected mother opossum to joey has been shown (*19*,*20*); those observations suggest alternative parasite transmission pathways within wildlife. 

Infection among companion animals, such as domestic and working canines and felines, has also been demonstrated throughout the United States (*6*). Dogs exposed to *T. cruzi* have been found in 23 states, as well as in Washington, DC, and the US Virgin Islands, although dogs infected in northern states likely reflect travel from regions where vectors are present (*16*,*20*). In Texas, the only state where Chagas disease in animals has been a reportable condition, 431 canine cases were reported during 2013–2015 (in addition to cases in 2 cats, 1 horse, 1 rat, 3 chimpanzees, and 1 walrus) (https://www.dshs.texas.gov/notifiable-conditions/zoonosis-control/zoonosis-control-diseases-and-conditions/chagas-disease/chagas-disease-data). After that period of widespread documenting of canine infections, the reporting requirement ceased, in part because of the substantial resources required to collate reports. Canine Chagas disease has been most studied in Texas, where cross-sectional and cohort studies have shown a prevalence ranging from ≈10% to >50% and a study across several large dog kennels showed an incidence of 30.7 new infections/100 dogs/year (e.g., *21*,*22*). Canines are a major domestic reservoir of *T. cruzi* parasites among communities in Latin America where human infection is routinely demonstrated (*23*). That link has also been shown in Texas communities located along the Rio Grande River, where infected canines and humans have been documented, raising additional concerns regarding ongoing domestic *T. cruzi* transmission (*24*–*27*).

*T. cruzi* infection among zoo-housed, exotic mammals has been recognized in states known to have triatomines, including Georgia, Alabama, Kansas, and Texas (*6*). In addition, infections occur in nonhuman primates at biomedical research facilities across the southern United States, posing challenges for research with those animal models (*28*). Exact transmission routes to these animals are hard to determine, but transmission likely occurs by ingesting the triatomine bug (*29*). Although many exotic animals have extensive travel histories, local infections are possible when triatomine are present on the premises.

## Autochthonous Human Chagas Disease in the United States

Autochthonous human *T. cruzi* infections have been identified in 8 states: California, Arizona, Texas, Tennessee, Louisiana, Missouri, Mississippi, and Arkansas (*8*). A systematic literature review found 29 confirmed and 47 suspected cases of locally acquired Chagas disease during 2000–2018; shared risk factors included rural residence, history of hunting or camping, and agricultural or outdoor work (*30*). Those numbers likely greatly underrepresent underlying human infections. The Council of State and Territorial Epidemiologists created a surveillance case definition for *T. cruzi* infection and Chagas disease in June 2024 (*31*); however, human Chagas disease is not a nationally notifiable disease, and thus the true prevalence or incidence of autochthonous Chagas disease remains unknown. Human Chagas disease is a notifiable disease in 8 states (Arizona, Arkansas, Louisiana, Mississippi, Tennessee, Texas, Utah, and Washington) and 2 California health jurisdictions (San Diego County and Los Angeles County).

**Figure 2:**
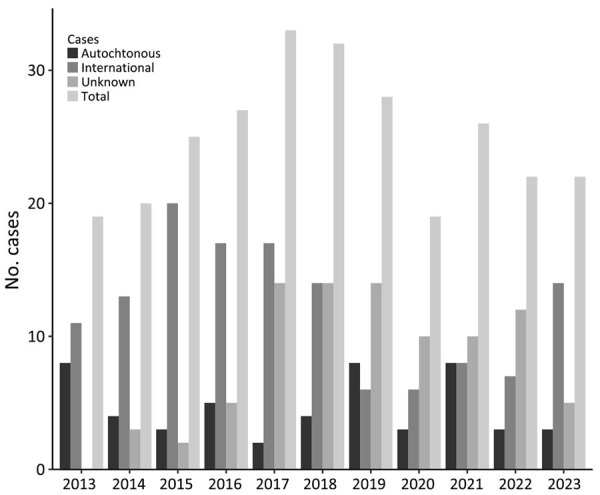
Yearly reported cases of autochthonous human Chagas disease in Texas in assessment of Chagas disease as endemic to the United States. Cases have been continuously reported with no apparent temporal trend (z-score −1.0004; p = 0.31), 2013–2023.

Texas has undertaken extensive efforts to document human Chagas disease; cases were first made reportable in the state in 2013 (https://www.dshs.texas.gov/notifiable-conditions/zoonosis-control/zoonosis-control-diseases-and-conditions/chagas-disease/chagas-disease-data). The first known autochthonous case of human Chagas disease in the United States occurred in an infant in 1955 in Corpus Christi, Texas, in a home known to be infested with triatomines (*32*). However, parasite transmission to humans in the region has occurred since prehistoric times, given, for example, a paleoparasitology study that recovered *T. cruzi* DNA in a mummified body (dated to 1,150 BP) of a man from western Texas with signs of megacolon (*33*). During 2013–2023, the Texas Department of State Health Services documented 50 probable and confirmed cases that were considered autochthonous, either because the area of vector exposure was known or because of a lack of travel to or previous residence in Chagas disease–endemic areas of Latin America (https://www.dshs.texas.gov/notifiable-conditions/zoonosis-control/zoonosis-control-diseases-and-conditions/chagas-disease/chagas-disease-data) (Figure 2; Appendix). Of those 50 cases, 3 were diagnosed at the acute stage, 44 at the chronic asymptomatic (indeterminate) stage, and 3 at the chronic symptomatic (determinate) stage. Based on CDC guidelines, diagnosis of chronic Chagas disease requires positive results by >2 tests that detect antibodies to different antigens, because no single test is sufficiently sensitive and specific for diagnosis (https://www.cdc.gov/chagas/index.html). Of the 47 reported chronic cases, 31 (66%) were confirmed with serologic testing at CDC. Of the 3 acute cases, 2 were acquired in central Texas (Austin–Round Rock metropolitan area); the third case was acquired in the Rio Grande Valley region of South Texas. A Spearman correlation test indicated that there was no temporal trend in the reported cases by year (z-score −1.0004; p = 0.31), underscoring that locally acquired cases are a stable threat in the state.

Triatomine species from the southwestern United States have been shown to have a longer postfeeding defecation behavior than more efficient triatomine vectors in Latin America (*34*); that behavior has been posed to reduce the risk for contact between infectious *T. cruzi* and the host. That narrative has contributed to the perception that triatomine species in the United States are not capable of stercorarian transmission. However, a study of *T. gerstaeckeri* and *T. sanguisuga* bugs documented simultaneous feeding and defecation by the 2 key North American vectors, although the measured postfeeding defecation indices were longer than those of the *Rhodnius Prolixus* kissing bug, a highly competent triatomine from South America (*35*). Although many of the autochthonous Chagas disease cases in the United States involve persons exposed to triatomines, additional case-patients report no exposure to triatomines (*36*,*37*), underscoring the cryptic nature of the vectors and suggesting alternative transmission routes should be considered (*8*). 

Oral transmission has been documented in certain regions of Latin America where fruit-based food or drink products have been contaminated with *T. cruzi* from triatomines living on trees (*38*). Triatomines in the United States have not been reported to inhabit fruit trees, and oral transmission is likely less relevant for explaining human cases acquired in the United States; nonetheless, oral parasite transmission through consumption of infected triatomines is speculated to be the primary mode of transmission to dogs (*39*). In addition, screening programs to detect potential congenital *T. cruzi* transmission in the United States are critical for preventing long-term sequelae of the disease (*40*).

## Definitions of Endemicity

The CDC defines endemic as the constant presence/usual prevalence of a disease or infectious agent in a population within a geographic area (*41*). In its characterization of infectious disease occurrence patterns, Clay’s Handbook of Environmental Health identifies an endemic pattern as “when an infection is always present at low or moderate levels within a given geographic area or defined population” (*42*). A “hyperendemic pattern,” the authors add, “is observed when infection occurs at high levels and affects all age groups equally” (*42*). WHO provides a more nuanced definition of specific terminology for malaria, such as endemic being “an area in which there is an ongoing, measurable incidence of malaria infection and mosquito-borne transmission over a succession of years”; the organization also includes specific subcategories for the percent of the population with malaria: “hypoendemic (0–10%), mesoendemic (10–50%), hyperendemic (constantly >50%), and holoendemic (constantly >75%)” (*43*). Such an operative scale for Chagas disease has not yet been developed, but acknowledging the nuanced nature of endemicity (as stated by WHO terminology) and the unique characteristics of Chagas disease occurrence in different regions will be crucial when considering the endemicity status of Chagas disease in the United States. The context in the southern United States presents well-established enzootic cycles and sporadic albeit constant locally acquired human cases (Figure 2), supporting a Chagas disease–endemic disease status.

## Consequences of Nonendemic Label

The current classification of the United States as nonendemic for Chagas disease has led to critical issues such as low physician and veterinary awareness of possible human and animal exposure to *T. cruzi* (*4*,*44*,*45*), which prevents appropriate differential diagnosis and could subsequently contribute to potential underreporting. The nonendemic label is coupled with the portrayal of Chagas disease as an essentially foreign or an exclusively travel-related issue in the media. Such misrecognition impedes effective disease management and underscores the need to reevaluate Chagas disease’s endemic status.

We propose that Chagas disease in the United States be classified as endemic and, more specifically, hypoendemic, acknowledging its presence and effects while emphasizing the need for heightened awareness and surveillance. We recognize the burden of locally acquired human disease in the United States does not approach the levels seen in some regions of Latin America but hope that labeling the United States as Chagas disease–endemic will also raise awareness for this neglected disease across its endemic range. This reclassification reflects a broader understanding of epidemiology that aligns with a One Health approach, recognizing the interconnectedness of human, animal, and environmental health. It also acknowledges the United States’ foundational and ongoing dependence on the highly variable modes of human migration and settlement. By incorporating ecologic, social, and geographic relationships, this shift paves the way for expanding research and intervention strategies. The United States contributes 23.3% of the world’s scientific research on Chagas disease, but most of it is focused on pharmacological and diagnostics development and immunology (*46*). Social and epidemiologic research, which should focus on populations that are disproportionately affected (*46*), is lacking. Recognizing Chagas disease as endemic to the United States would ideally help increase funding agencies’ investment in research toward improved diagnostics and treatment and, perhaps more critically, would support local public health agencies in obtaining resources needed to educate communities, report cases, and prevent new infections. Last, from a global health perspective, without recognizing stable transmission within its borders, the United States will be unable to reach its Sustainable Development Goals outlined in the WHO initiative Ending the Neglect to Attain Sustainable Development Goals: A Road Map for Neglected Tropical Disease 2021–2030; specifically, the third foundational pillar that centers on changing “operating models and culture to facilitate country ownership” would be unattainable (https://www.paho.org/en/topics/chagas-disease).

## Conclusions

*T. cruzi* and the ecologic conditions that sustain its transmission cycles are naturally occurring throughout the southern half of the United States. Infection has been consistently demonstrated in wildlife reservoirs, companion animals, zoo and exotic mammals, and humans. At least 4 triatomine species are frequently encountered in homes and found to be harboring *T. cruzi* parasites. Canine Chagas disease is a concern in many working and companion dog populations in the southern United States but is likely underrecognized in many areas. The exposure of nonhuman primates to *T. cruzi*–infected triatomines poses a challenge to medical research. Moreover, the lack of reporting requirements for human Chagas disease adds complexity to the documentation of autochthonous cases. The number of documented autochthonous cases is higher in Texas than in other states, and cases are consistently documented each year.

This body of evidence justifies recognizing that Chagas disease is endemic to the United States, and not just from a veterinary perspective. Updating Chagas disease endemicity status as hypoendemic is a crucial step toward a more effective management model, one that addresses the unique challenges and complexities of this country regarding vectorborne diseases. Such a shift will help reform curriculum in professional schools to enable the next generation of practitioners to be competent in recognizing the low but present risk for locally acquired *T. cruzi* infections and better serve those who acquire the parasite elsewhere and require diagnosis in the United States. There is an opportunity to learn from the public health experiences in Mexico and other regions of Latin America that have long faced a high burden of human Chagas disease; those experiences call for increased understanding and disease management that integrates biomedical, sociocultural and policy perspectives (*47*). Managing endemic Chagas disease in the United States will require overcoming systemic, structural, clinical, and psychosocial healthcare barriers (*48*). Case investigations would ideally have standardized data collection and case classification, to include travel history, triatomine encounters, and proximity to suitable triatomine habitat, all of which are useful in assessing local transmission risk (*49*). In the meantime, solutions for Chagas disease can be advanced through the study of the unfortunate abundance of naturally infected animals across the southern United States (*50*). To achieve the Sustainable Development Goals for the 2030 Neglected Tropical Disease roadmap, recognizing Chagas disease endemicity in the United States as a regional issue will be imperative to begin implementing local, state, and national strategic plans to tackle this neglected disease that, as has been demonstrated, has never been exclusively tropical.

Appendix Additional information about Chagas disease as an endemic disease in the United States
