# Supplementary material for: Chagas Disease, an Endemic Disease in the United States
Source: Emerg Infect Dis. 2025 Sep;31(9):1691–7. doi: 10.3201/eid3109.241700 (PMC12407112; doi:10.3201/eid3109.241700)
Supplement: Appendix — Additional information about Chagas disease as an endemic disease in the United States [file 24-1700-Techapp-s1.pdf]

Article DOI: https://doi.org/10.3201/eid3109.241700

EID cannot ensure accessibility for supplementary materials supplied by authors. Readers who have difficulty accessing supplementary content should contact the authors for assistance.

Chagas Disease, an Endemic Disease in the United States

Appendix

Appendix Table. All states with compiled references to show occurrence of triatomines, infected animals, and autochthonous human Chagas disease

State	Documented kissing bugs, yes/no	References	Wildlife, domestic, or captive animal *T. cruzi* Infection, yes/no	References	Autochthonous Chagas disease in humans, yes/no	References
Alabama	Yes	(*1*–*3*)	Yes	(*2*,*4*)	No	
Arizona	Yes	(*1*,*3*,*5*–*10*)	Yes	(*4*,*11*,*12*)	Yes	(*13*,*14*)
Arkansas	Yes	(*1*,*3*)	No		Yes	(*14*)
California	Yes	(*1*,*3*,*5*,*8*,*15*–*19*)	Yes	(*4*,*11*,*12*,*20*–*26*)	Yes	(*25*,*27*,*28*)
Connecticut	No		No		No	
Colorado	Yes	(*1*,*3*,*5*)	No		No	
Delaware	Yes	(*1*)	No		No	
Florida	Yes	(*1*,*3*,*29*)	Yes	(*4*,*12*,*30*–*33*)	No	
Georgia	Yes	(*1*,*3*,*34*–*36*)	Yes	(*4*,*12*,*30*,*32*,*35*–*39*)	No	
Idaho	No		No		No	
Illinois	Yes	(*1*,*3*)	No		No	
Indiana	Yes	(*1*,*3*)	No		No	
Iowa	No		No		No	
Kansas	Yes	(*1*,*3*,*40*,*41*)	Yes	(*42*)	No	
Kentucky	Yes	(*1*,*3*)	Yes	(*4*,*43*)	No	
Louisiana	Yes	(*1*,*3*,*44*)	Yes	(*4*,*45*,*46*)	Yes	(*4*,*44*,*47*)
Maine	No		No		No	
Maryland	Yes	(*1*,*3*)	Yes	(*4*,*48*,*49*)	No	
Massachusetts	No		No		No	
Michigan	No		No		No	
Minnesota	No		No		No	
Mississippi	Yes	(*1*,*3*,*50*)	No		Yes	(*50*–*52*)
Missouri	Yes	(*1*,*3*)	Yes	(*12*)	Yes	(*53*)
Montana	No		No		No	
Nebraska	Yes	(*1*)	No		No	
Nevada	Yes	(*5*)	No		No	
New Hampshire	No		No		No	
New Jersey	Yes	(*1*,*4*)	No		No	
New Mexico	Yes	(*1*,*3*,*5*,*54*–*56*)	Yes	(*4*,*56*)	No	
New York	No		No		No	
North Carolina	Yes	(*1*,*3*)	Yes	(*4*,*57*)	No	
North Dakota	No		No		No	
Ohio	Yes	(*1*,*3*)	No		No	
Oklahoma	Yes	(*1*,*3*,*58*)	Yes	(*4*,*59*)	No	
Oregon	No		No		No	
Pennsylvania	Yes	(*1*,*3*)	No		No	
Rhode Island	No		No		No	
South Carolina	Yes	(*1*,*3*,*36*)	Yes	(*36*,*60*)	No	
South Dakota	No		No		No	
Tennessee	Yes	(*1*,*3*,*61*)	Yes	(*4*,*61*)	Yes	(*61*)
Texas	Yes	(*1*,*3*,*10*,*55*,*62*–*70*)	Yes	(*4*,*32*,*62*,*66*,*68*,*71*–*75*)	Yes	(*4*,*67*,*76*–*86*)
Utah	Yes	(*1*,*3*)	No		No	

State	Documented kissing bugs, yes/no	References	Wildlife, domestic, or captive animal *T. cruzi* Infection, yes/no	References	Autochthonous Chagas disease in humans, yes/no	References
Virginia	Yes	(*1*,*3*)	Yes	(*4*,*12*,*87*)	No	
Vermont	No		No		No	
Washington	No		No		No	
West Virginia	Yes	(*1*)	No		No	
Wisconsin	No		No		No	
Wyoming	Yes	(*88*)	No		No	

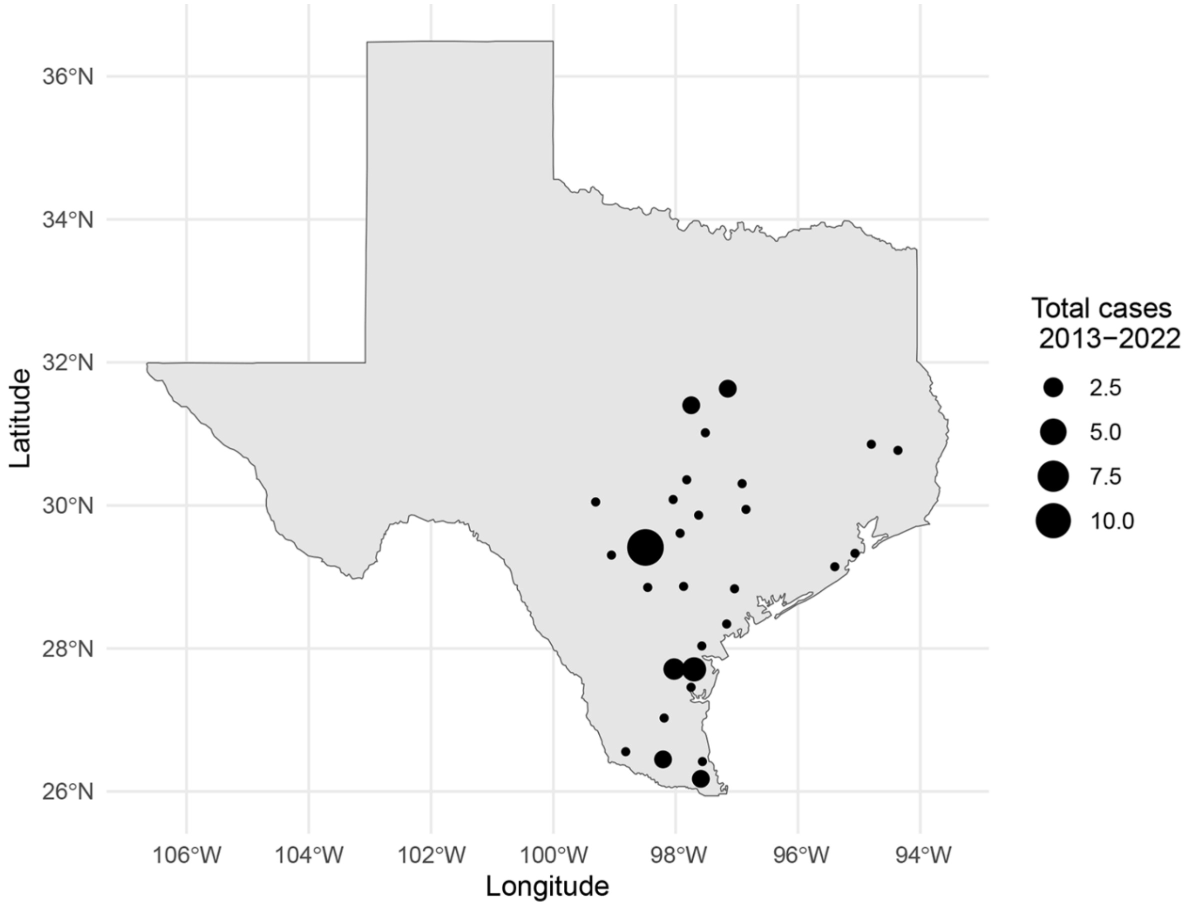

Appendix Figure. Texas map showing the number of autochthonous human Chagas disease cases by country in which the shape of each circle is proportional to the number of human cases, 2013-2022

References

1. Usinger RL. The Triatominae of North and Central America and the West Indies and their public health significance. Washington (DC): US Government Printing Office; 1944.

2. Olsen PF, Shoemaker JP, Turner HF, Hays KL. Incidence of *Trypanosoma cruzi* (Chagas) in wild vectors and reservoirs in East-Central Alabama. J Parasitol. 1964;50:599–603. PubMed https://doi.org/10.2307/3276112

3. Lent H, Wygodzinsky P. Revision of the Triatominae (Hemiptera, Reduviidae) and their significance as vectors of Chagas' disease. Bull Am Mus Nat Hist. 1979;163:123–520.

4. Bern C, Kjos S, Yabsley MJ, Montgomery SP. *Trypanosoma cruzi* and Chagas' disease in the United States. Clin Microbiol Rev. 2011;24:655–81. PubMed https://doi.org/10.1128/CMR.00005-11

5. Ryckman RE. The kissing bug problem in western North America. Bull Soc Vector Ecol. 1981;6:167–9.

6. Reisenman CE, Lawrence G, Guerenstein PG, Gregory T, Dotson E, Hildebrand JG. Infection of kissing bugs with *Trypanosoma cruzi*, Tucson, Arizona, USA. Emerg Infect Dis. 2010;16:400–5. PubMed https://doi.org/10.3201/eid1603.090648

7. Wood SF. Observations on vectors of Chagas' disease in the United States. II. Arizona. Am J Trop Med. 1943;23:315–20. https://doi.org/10.4269/ajtmh.1943.s1-23.315

8. Klotz JH, Dorn PL, Logan JL, Stevens L, Pinnas JL, Schmidt JO, et al. "Kissing bugs": potential disease vectors and cause of anaphylaxis. Clin Infect Dis. 2010;50:1629–34. PubMed https://doi.org/10.1086/652769

9. Ryckman RE. The vertebrate hosts of the Triatominae of North and Central America and the West Indies (Hemiptera: Reduviidae: Triatominae). Bull Soc Vector Ecol. 1986;11:221–41.

10. Wood SF. Notes on the distribution and habits of reduviid vectors of Chagas' disease in the southwestern United States (Hemiptera, Reduviidae). Pan-Pac Entomol. 1941;•••:17.

11. Wood SF. Mammal blood parasite records from southwestern United States and Mexico. J Parasitol. 1952;38:85–6. https://doi.org/10.2307/3274183

12. Brown EL, Roellig DM, Gompper ME, Monello RJ, Wenning KM, Gabriel MW, et al. Seroprevalence of *Trypanosoma cruzi* among eleven potential reservoir species from six states across the southern United States. Vector Borne Zoonotic Dis Larchmt N. 2010;10:757–63.

13. Harris N, Woc-Colburn L, Gunter SM, Gorchakov R, Murray KO, Rossmann S, et al. Autochthonous Chagas disease in the southern United States: a case report of suspected residential and military exposures. Zoonoses Public Health. 2017;64:491–3. PubMed https://doi.org/10.1111/zph.12360

14. Lynn MK, Bossak BH, Sandifer PA, Watson A, Nolan MS. Contemporary autochthonous human Chagas disease in the USA. Acta Trop. 2020;205:105361. PubMed https://doi.org/10.1016/j.actatropica.2020.105361

15. Mortenson EW, Walsh JD. Review of the *Triatoma protracta* problem in the Sierra Nevada foothills of California. Proceedings of the 31st Annual Conference of the California Mosquito Control Association California Mosquito Control Association; 1963, Sacramento, CA. p. 44–5.

16. Walsh JD, Jones JP. Public health significance of the cone-nosed bug, *Triatoma protracta* (Uhler), in the Sierra Nevada foothills of California. Calif Vector Views. 1962;9:33–7.

17. Mehringer PJ Jr, Wood SF. A resampling of wood rat houses and human habitations in Griffith Park, Los Angeles, for *Triatoma protracta* and *Trypanosoma cruzi.* Bull South Calif Acad Sci. 2022;57:39–46.

18. Wood SF, Wood FD. Nocturnal aggregation and invasion of homes in southern California by insect vectors of Chagas' disease. J Econ Entomol. 1964;57:775–6. https://doi.org/10.1093/jee/57.5.775

19. Kofoid CA, Mcculloch I. On *Trypanosoma triatomae*, a new flagellate from a hemipteran bug from the nests of the wood rat *Neotoma fuscipes.* Univ Calif Publ Zool. 1916;•••:16.

20. Wood SF. Blood parasites of mammals of the Californian Sierra Nevada foothills, with special reference to *Trypanosoma cruzi* Chagas and *Hepatozoon leptosoma* sp. n. Bull South Calif Acad Sci. 1962;61:161–76.

21. Wood SF. *Trypanosoma cruzi*: new foci of enzootic Chagas' disease in California. Exp Parasitol. 1975;38:153–60. PubMed https://doi.org/10.1016/0014-4894(75)90017-X

22. Wood SF, Wood FD. Ecological relationships of *Triatoma p. protracta* (Uhler) in Griffith Park, Los Angeles, Calif. Pac Insects. 1967;•••:9.

23. Wood SF. An additional California locality for *Trypanosoma cruzi* Chagas in the western cone-nosed bug, *Triatoma protracta* (Uhler). J Parasitol. 1944;30:199. https://doi.org/10.2307/3272802

24. Ryan CP, Hughes PE, Howard EB. American trypanosomiasis (Chagas' disease) in a striped skunk. J Wildl Dis. 1985;21:175–6. PubMed https://doi.org/10.7589/0090-3558-21.2.175

25. Navin TR, Roberto RR, Juranek DD, Limpakarnjanarat K, Mortenson EW, Clover JR, et al. Human and sylvatic *Trypanosoma cruzi* infection in California. Am J Public Health. 1985;75:366–9. PubMed https://doi.org/10.2105/AJPH.75.4.366

26. Valdez-Tah A, Ibarra-Cerdeña CN. Call to action: a literature review of Chagas disease risk in California 1916-2018. PLoS Negl Trop Dis. 2021;15:e0009035. PubMed https://doi.org/10.1371/journal.pntd.0009035

27. Hernandez S, Flores CA, Viana GM, Sanchez DR, Traina MI, Meymandi SK. Autochthonous transmission of *Trypanosoma cruzi* in southern California. Open Forum Infect Dis. 2016;3:ofw227. PubMed https://doi.org/10.1093/ofid/ofw227

28. Schiffler RJ, Mansur GP, Navin TR, Limpakarnjanarat K. Indigenous Chagas' disease (American trypanosomiasis) in California. JAMA. 1984;251:2983–4. PubMed https://doi.org/10.1001/jama.1984.03340460061025

29. Packchanian A. Experimental transmission of *Trypanosoma cruzi* infection in animals by *Triatoma sanguisuga ambigua.* Public Health Rep. 1940;55:1526–32. https://doi.org/10.2307/4583416

30. McKeever S, Gorman GW, Norman L. Occurrence of a *Trypanosoma cruzi*-like organism in some mammals from southwestern Georgia and northwestern Florida. J Parasitol. 1958;44:583–7. PubMed https://doi.org/10.2307/3274538

31. Telford SR Jr, Forrester DJ. Hemoparasites of raccoons (*Procyon lotor*) in Florida. J Wildl Dis. 1991;27:486–90. PubMed https://doi.org/10.7589/0090-3558-27.3.486

32. Schaffer GD, Hanson WL, Davidson WR, Nettles VF. Hematotropic parasites of translocated raccoons in the southeast. J Am Vet Med Assoc. 1978;173:1148–51. PubMed https://doi.org/10.2460/javma.1978.173.09.1148

33. Torhorst CW, Ledger KJ, White ZS, Milleson MP, Corral CC, Beatty NL, et al. *Trypanosoma cruzi* infection in mammals in Florida: new insight into the transmission of *T. cruzi* in the southeastern United States. Int J Parasitol Parasites Wildl. 2023;21:237–45. PubMed https://doi.org/10.1016/j.ijppaw.2023.06.009

34. LeConte JL, Retzius A. September 25th; descriptions of new species of *Astacus* from Georgia; on a new species of *Gelasimus*; remarks on two species of American cimex; on artificially formed skulls from the ancient world. Proc Acad Nat Sci Philadelphia. 1855;7:399–408.

35. Pung OJ, Spratt J, Clark CG, Norton TM, Carter J. *Trypanosoma cruzi* infection of free-ranging lion-tailed macaques (*Macaca silenus*) and ring-tailed lemurs (*Lemur catta*) on St. Catherine's Island, Georgia, USA. J Zoo Wildl Med. 1998;29:25–30. PubMed

36. Yabsley MJ, Noblet GP. Seroprevalence of *Trypanosoma cruzi* in raccoons from South Carolina and Georgia. J Wildl Dis. 2002;38:75–83. PubMed https://doi.org/10.7589/0090-3558-38.1.75

37. Parrish EA, Mead AJ. Determining the prevalence of *Trypanosoma cruzi* in road-killed opossums (*Didelphis virginiana*) from Baldwin County, Georgia, using polymerase chain reaction. Ga J Sci. 2010;68:132–40.

38. Pietrzak SM, Pung OJ. Trypanosomiasis in raccoons from Georgia. J Wildl Dis. 1998;34:132–6. PubMed https://doi.org/10.7589/0090-3558-34.1.132

39. Pung OJ, Banks CW, Jones DN, Krissinger MW. *Trypanosoma cruzi* in wild raccoons, opossums, and triatomine bugs in southeast Georgia, U.S.A. J Parasitol. 1995;81:324–6. PubMed https://doi.org/10.2307/3283947

40. Kimball BS. *Conorhinus sanguisugus*: its habits and life history. Trans Ann Mtg Kansas Acad Sci. 1893-1894;14:128–31. https://doi.org/10.2307/3623926

41. Grundemann AW. Studies on the biology of *Triatoma sanguisuga* (Leconte) in Kansas, (Reduviidae, Hemiptera). J Kans Entomol Soc. 1947;20:77–85.

42. Huckins GL, Eshar D, Schwartz D, Morton M, Herrin BH, Cerezo A, et al. *Trypanosoma cruzi* infection in a zoo-housed red panda in Kansas. J Vet Diagn Invest. 2019;31:752–5. PubMed https://doi.org/10.1177/1040638719865926

43. Groce B. *Trypanosoma cruzi* in wild raccoons and opossums from Kentucky [cited 2024 Oct 15]. https://digitalcommons.wku.edu/theses/31

44. Dorn PL, Perniciaro L, Yabsley MJ, Roellig DM, Balsamo G, Diaz J, et al. Autochthonous transmission of *Trypanosoma cruzi*, Louisiana. Emerg Infect Dis. 2007;13:605–7. PubMed https://doi.org/10.3201/eid1304.061002

45. Yaeger RG. The prevalence of *Trypanosoma cruzi* infection in armadillos collected at a site near New Orleans, Louisiana. Am J Trop Med Hyg. 1988;38:323–6. PubMed https://doi.org/10.4269/ajtmh.1988.38.323

46. Barr SC, Brown CC, Dennis VA, Klei TR. The lesions and prevalence of *Trypanosoma cruzi* in opossums and armadillos from southern Louisiana. J Parasitol. 1991;77:624–7. PubMed https://doi.org/10.2307/3283170

47. Louisiana Office of Public Health. Chagas disease–American trypanosomiasis. Baton Rouge (LA): The Office; 2018.

48. Walton BC, Bauman PM, Diamond LS, Herman CM. The isolation and identification of *Trypanosoma cruzi* from raccoons in Maryland. Am J Trop Med Hyg. 1958;7:603–10. PubMed https://doi.org/10.4269/ajtmh.1958.7.603

49. Herman CM, Bruce JI Jr. Occurrence of *Trypanosoma cruzi* in Maryland. Proc Helminthol Soc Wash. 1962;29:55–8.

50. Cantey PT, Hand S, Currier M, Jett P, Montgomery SP. Chagas disease in Mississippi: investigation of suspected autochthonous infections in the United States. Abstract H3. 2008 International Conference on Emerging Infectious Diseases; Mar 16–19; Atlanta, GA.

51. Mississippi State Department of Health. Chagas disease in Mississippi. Mississippi Morbidity Report. 2008;24:1–2.

52. Cantey PT, Stramer SL, Townsend RL, Kamel H, Ofafa K, Todd CW, et al. The United States *Trypanosoma cruzi* Infection Study: evidence for vector-borne transmission of the parasite that causes Chagas disease among United States blood donors. Transfusion. 2012;52:1922–30. PubMed https://doi.org/10.1111/j.1537-2995.2012.03581.x

53. Turabelidze G, Vasudevan A, Rojas-Moreno C, Montgomery SP, Baker M, Pratt D, et al. Autochthonous Chagas disease–Missouri, 2018. MMWR Morb Mortal Wkly Rep. 2020;69:193–5. PubMed https://doi.org/10.15585/mmwr.mm6907a4

54. Ryckman RE. The Triatominae of North and Central America and the West Indies: a checklist with synonymy (Hemiptera: Reduviidae: Triatominae). Bull Soc Vector Ecol. 1984;9:71–83.

55. Pippin WF, Law PF, Gaylor MJ. *Triatoma sanguisuga* Texana Usinger and *Triatoma sanguisuga* indictive Neiva naturally infected with *Trypanosoma cruzi* Chagas in Texas (Hemiptera: Triatominae) (Kinetoplastida: Trypanosomidae). J Med Entomol. 1968;5:134. PubMed https://doi.org/10.1093/jmedent/5.1.134

56. Wood SF, Wood FD. Observations on vectors of Chagas' disease in the United States. III. New Mexico. Am J Trop Med Hyg. 1961;10:155–65. PubMed https://doi.org/10.4269/ajtmh.1961.10.155

57. Karsten V, Davis C, Kuhn R. *Trypanosoma cruzi* in wild raccoons and opossums in North Carolina. J Parasitol. 1992;78:547–9. PubMed https://doi.org/10.2307/3283667

58. Griffith MB. The bloodsucking conenose, or "big bedbug," *Triatoma sanguisuga* (Leconte), in an Oklahoma City household. Proc Okla Acad Sci. 1948;28:24–7.

59. John DT, Hoppe KL. *Trypanosoma cruzi* from wild raccoons in Oklahoma. Am J Vet Res. 1986;47:1056–9. PubMed https://doi.org/10.2460/ajvr.1986.47.05.1056

60. Rosypal AC, Tidwell RR, Lindsay DS. Prevalence of antibodies to *Leishmania infantum* and *Trypanosoma cruzi* in wild canids from South Carolina. J Parasitol. 2007;93:955–7. PubMed https://doi.org/10.1645/GE-1057R.1

61. Herwaldt BL, Grijalva MJ, Newsome AL, McGhee CR, Powell MR, Nemec DG, et al. Use of polymerase chain reaction to diagnose the fifth reported US case of autochthonous transmission of *Trypanosoma cruzi,* in Tennessee, 1998. J Infect Dis. 2000;181:395–9. PubMed https://doi.org/10.1086/315212

62. Kjos SA, Snowden KF, Craig TM, Lewis B, Ronald N, Olson JK. Distribution and characterization of canine Chagas disease in Texas. Vet Parasitol. 2008;152:249–56. PubMed https://doi.org/10.1016/j.vetpar.2007.12.021

63. Packchanian A. Natural infection of *Triatoma gerstakeri* with *Trypanosoma cruzi* in Texas. Public Health Rep. 1939;54:1547–54. https://doi.org/10.2307/4582999

64. Lathrop GD, Ominsky AJ. Chagas' disease study in a group of individuals bitten by North American triatomids. Aeromed Rev. 1965;9:1–5.PubMed

65. Beard CB, Pye G, Steurer FJ, Rodriguez R, Campman R, Peterson AT, et al. Chagas disease in a domestic transmission cycle, southern Texas, USA. Emerg Infect Dis. 2003;9:103–5. PubMed https://doi.org/10.3201/eid0901.020217

66. Eads RB, Trevino HA, Campos EG. Triatoma (Hemiptera: Reduviidae) infected with *Trypanosoma cruzi* in South Texas wood rat dens. Southwest Nat. 1963;8:38–42. https://doi.org/10.2307/3669426

67. Kjos SA, Snowden KF, Olson JK. Biogeography and *Trypanosoma cruzi* infection prevalence of Chagas disease vectors in Texas, USA. Vector Borne Zoonotic Dis. 2009;9:41–50. PubMed https://doi.org/10.1089/vbz.2008.0026

68. Burkholder JE, Allison TC, Kelly VP. *Trypanosoma cruzi* (Chagas) (Protozoa: Kinetoplastida) in invertebrate, reservoir, and human hosts of the lower Rio Grande valley of Texas. J Parasitol. 1980;66:305–11. PubMed https://doi.org/10.2307/3280824

69. Packchanian A. Infectivity of the Texas strain of *Trypanosoma cruzi* to man. Am J Trop Med. 1943;s1-23:23. https://doi.org/10.4269/ajtmh.1943.s1-23.309

70. Wood SF. New localities for *Trypanosoma cruzi* Chagas in southwestern United States. Am J Epidemiol. 1941;34:1–13. https://doi.org/10.1093/oxfordjournals.aje.a118745

71. Williams GD, Adams LG, Yaeger RG, McGrath RK, Read WK, Bilderback WR. Naturally occurring trypanosomiasis (Chagas' disease) in dogs. J Am Vet Med Assoc. 1977;171:171–7. PubMed https://doi.org/10.2460/javma.1977.171.02.171

72. Centers for Disease Control and Prevention. Using museum collections to detect pathogens. [cited 2024 Mar 10]. https://stacks.cdc.gov/view/cdc/18015

73. Grögl M, Kuhn RE, Davis DS, Green GE. Antibodies to *Trypanosoma cruzi* in coyotes in Texas. J Parasitol. 1984;70:189–91. PubMed https://doi.org/10.2307/3281962

74. Packchanian A. Reservoir hosts of Chagas' disease in the State of Texas. Natural infection of nine-banded armadillo (*Dasypus novemcinctus texanus*), house mice (*Mus musculus*), opossum (*Didelphis virginiana*), and wood rats (*Neotoma micropus micropus*), with *Trypanosoma cruzi* in the state of Texas. Am J Trop Med. 1942;•••:22.

75. Hanford EJ, Zhan FB, Lu Y, Giordano A. Chagas disease in Texas: recognizing the significance and implications of evidence in the literature. Soc Sci Med. 2007;65:60–79. PubMed https://doi.org/10.1016/j.socscimed.2007.02.041

76. Curtis-Robles R, Zecca IB, Roman-Cruz V, Carbajal ES, Auckland LD, Flores I, et al. *Trypanosoma cruzi* (agent of Chagas disease) in sympatric human and dog populations in "colonias" of the Lower Rio Grande Valley of Texas. Am J Trop Med Hyg. 2017;96:805–14. PubMed https://doi.org/10.4269/ajtmh.16-0789

77. Texas Department of State Health Services. Chagas disease data [cited 2024 Mar 7]. https://www.dshs.texas.gov/chagas-disease/chagas-disease-data

78. Walker DH. Fatal cardiac failure in a 7-month-old child. Pediatr Dev Pathol. 2003;6:582–4. PubMed https://doi.org/10.1007/s10024-003-1234-7

79. Leiby DA, Rentas FJ, Nelson KE, Stambolis VA, Ness PM, Parnis C, et al. Evidence of *Trypanosoma cruzi* infection (Chagas' disease) among patients undergoing cardiac surgery. Circulation. 2000;102:2978–82. PubMed https://doi.org/10.1161/01.CIR.102.24.2978

80. Garcia MN, O'Day S, Fisher-Hoch S, Gorchakov R, Patino R, Feria Arroyo TP, et al. One Health interactions of Chagas disease vectors, canid hosts, and human residents along the Texas-Mexico border. PLoS Negl Trop Dis. 2016;10:e0005074. PubMed https://doi.org/10.1371/journal.pntd.0005074

81. Ochs DE, Hnilica VS, Moser DR, Smith JH, Kirchhoff LV. Postmortem diagnosis of autochthonous acute chagasic myocarditis by polymerase chain reaction amplification of a species-specific DNA sequence of *Trypanosoma cruzi.* Am J Trop Med Hyg. 1996;54:526–9. PubMed https://doi.org/10.4269/ajtmh.1996.54.526

82. Greer DA. Found: two cases of Chagas disease. Tex Health Bull. 1955;9.

83. Woody NC, Woody HB. American trypanosomiasis (Chagas' disease); first indigenous case in the United States. J Am Med Assoc. 1955;159:676–7. PubMed https://doi.org/10.1001/jama.1955.02960240042010a

84. Garcia MN, Aguilar D, Gorchakov R, Rossmann SN, Montgomery SP, Rivera H, et al. Evidence of autochthonous Chagas disease in southeastern Texas. Am J Trop Med Hyg. 2015;92:325–30. PubMed https://doi.org/10.4269/ajtmh.14-0238

85. Webber BJ, Wozniak EJ, Chang D, Bush KN, Wilson MC, Watts JA, et al. A case of Chagas cardiomyopathy following infection in South Central Texas [cited 2021 Apr 19]. https://apps.dtic.mil/sti/citations/AD1036440

86. Gunter SM, Murray KO, Gorchakov R, Beddard R, Rossmann SN, Montgomery SP, et al. Likely autochthonous transmission of *Trypanosoma cruzi* to humans, South Central Texas, USA. Emerg Infect Dis. 2017;23:500–3. PubMed https://doi.org/10.3201/eid2303.161157

87. Hancock K, Zajac AM, Pung OJ, Elvinger F, Rosypal AC, Lindsay DS. Prevalence of antibodies to *Trypanosoma cruzi* in raccoons (*Procyon lotor*) from an urban area of northern Virginia. J Parasitol. 2005;91:470–2. PubMed https://doi.org/10.1645/GE-399R

88. Reeves WK, Miller MM. A new state record for *Triatoma sanguisuga* (Leconte) (Hemiptera: Reduviidae) from Wyoming, U.S.A. Comp Parasitol. 2020;87:118. https://doi.org/10.1654/1525-2647-87.1.118
